# Rhizosphere assembly alters along a chronosequence in the Hallstätter glacier forefield (Dachstein, Austria)

**DOI:** 10.1093/femsec/fiae005

**Published:** 2024-01-25

**Authors:** Wisnu Adi Wicaksono, Maximilian Mora, Samuel Bickel, Christian Berg, Ingolf Kühn, Tomislav Cernava, Gabriele Berg

**Affiliations:** Institute of Environmental Biotechnology, Graz University of Technology, Graz 8010, Austria; Institute of Environmental Biotechnology, Graz University of Technology, Graz 8010, Austria; Institute of Environmental Biotechnology, Graz University of Technology, Graz 8010, Austria; Institute of Plant Sciences, University of Graz, Graz 8010, Austria; Department of Community Ecology, Helmholtz Centre for Environmental Research—UFZ, Halle 06120, Germany; Institute of Biology/Geobotany and Botanical Garden, Martin Luther University Halle–Wittenberg, Halle 06099, Germany; German Centre for Integrative Biodiversity Research (iDiv) Halle–Jena–Leipzig, Leipzig 04103, Germany; Institute of Environmental Biotechnology, Graz University of Technology, Graz 8010, Austria; Institute of Environmental Biotechnology, Graz University of Technology, Graz 8010, Austria; Leibniz Institute for Agricultural Engineering and Bioeconomy Potsdam (ATB), Potsdam 14469, Germany; Institute for Biochemistry and Biology, University of Potsdam, Potsdam 14469, Germany

**Keywords:** bacteria, deglaciation, glacier forefield, metagenome, microbiome, pioneer plants, soil microorganisms

## Abstract

Rhizosphere microbiome assembly is essential for plant health, but the temporal dimension of this process remains unexplored. We used a chronosequence of 150 years of the retreating Hallstätter glacier (Dachstein, Austria) to disentangle this exemplarily for the rhizosphere of three pioneer alpine plants. Time of deglaciation was an important factor shaping the rhizosphere microbiome. Microbiome functions, i.e. nutrient uptake and stress protection, were carried out by ubiquitous and cosmopolitan bacteria. The rhizosphere succession along the chronosequence was characterized by decreasing microbial richness but increasing specificity of the plant-associated bacterial community. Environmental selection is a critical factor in shaping the ecosystem, particularly in terms of plant-driven recruitment from the available edaphic pool. A higher rhizosphere microbial richness during early succession compared to late succession can be explained by the occurrence of cold-acclimated bacteria recruited from the surrounding soils. These taxa might be sensitive to changing habitat conditions that occurred at the later stages. A stronger influence of the plant host on the rhizosphere microbiome assembly was observed with increased time since deglaciation. Overall, this study indicated that well-adapted, ubiquitous microbes potentially support pioneer plants to colonize new ecosystems, while plant-specific microbes may be associated with the long-term establishment of their hosts.

## Introduction

The complex interplay between plant hosts and their rhizosphere microbiota is important for plant health (Cordovez et al. 2019, Berg et al. 2020, Trivedi et al. 2020). Interactions between plants and microorganisms have the potential to significantly contribute to plant adaptation, i.e. mediating host immunity, improving tolerance to environmental stress, facilitating access to new nutrient sources, and supporting resilience when exposed to specific environmental changes. They are collectively known as microbe-mediated adaptation (Petipas et al. 2021). All these processes are suggested to be influenced by plant genotype and the environment, including the extent of anthropogenic impacts on the ecosystem (Menzel et al. 2017, Kusstatscher et al. 2021, Berg and Cernava 2022, Cosme 2023). Plants assemble their rhizosphere microbiome by recruiting bacteria from seeds and the surrounding environment (Abdelfattah et al. 2021, Wicaksono et al. 2022). However, there are still several open questions regarding the complex interactions between plants and microbes, such as how much the plant itself assembles a microbial community from the surrounding soil and how this process is influenced by environmental changes.

Glaciers are model ecosystems of special interest due to their global relevance and accelerated retreat in the face of anthropogenic climate change. In glacier forefields, the successional age drives plant species composition, resulting in a gradient of increasing diversity and specificity within plant communities (Fickert et al. 2017, Ficetola et al. 2021). Moreover, the succession is driven by stochastic and deterministic processes. For plants, it is known that early successional species are rather generalists, and only later during succession, specialist species are found (Büchi and Vuilleumier 2014). Recently, glacier forefields were used to advance our understanding of the successional development of soil microbiomes (Tscherko et al. 2003, Bardgett et al. 2007). A study in a glacier forefield in the Austrian Alps showed that the soil microbial community was more closely related to plant communities than to environmental factors, supporting the notion that biotic factors are crucial in the successional assembly of diverse ecosystems (Junker et al. 2021). In contrast, He et al. (2023) tried to predict plant species composition from microbial composition and did not find a clear correlation between plant and microbiome assembly. Additionally, abiotic factors (i.e. physiochemical and microclimatic spatial variation at the site scale) shape bacterial community assembly during primary colonization (Rolli et al. 2022). However, the extent to which these factors play a role in the rhizosphere microbiome assembly is not well understood, especially during early succession.

Forefields of retreating glaciers provide an ideal setting to study the temporal dimension of rhizosphere microbiome assembly by space-for-time substitution and can provide insights into future shifts of rhizosphere microbiomes that may occur under changing environmental conditions (Bradley et al. 2014, Hotaling et al. 2017). Here, we investigated the succession of bacterial communities in the rhizosphere of three pioneering plant species in the forefield of the Hallstätter glacier (Austria). We used 16S rRNA gene amplicon sequencing and shotgun metagenomic sequencing to analyse the composition and function of microbiomes associated with *Papaver alpinum* L., *Hornungia alpina* (L.) O. Appel, and *Sedum atratum* L. The main objectives of this study were (i) to identify the adaptation of the functional potential associated with pioneer plant microbiomes during early succession after 10 years of deglaciation and (ii) to characterize bacterial compositional shifts in the soil and rhizosphere of the three pioneer plants where the glacier retreated 10, 70, and 150 years ago. Understanding successional shifts in microbiomes that are emerging in glacier forefields provides key insights into the consequences of future climate change regarding the dynamics of biodiversity and potential ecosystem functions.

## Materials and methods

### Sample collection and DNA extraction

**Figure 1.**
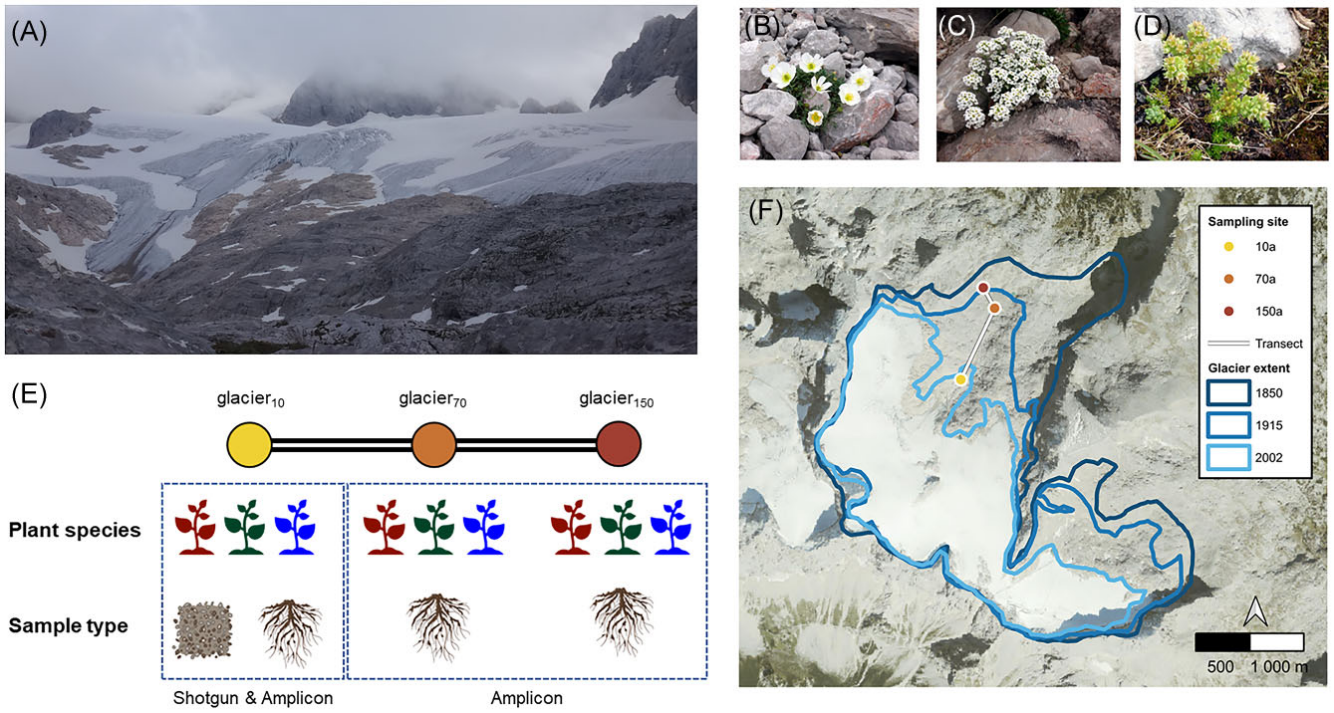
Topological profiles and the plant species naturally occurring at the studied sampling sites. (A) Representative picture of the Hallstätter glacier indicating deglaciation in this area. Examples of plants used for microbial community analysis are *P. alpinum* (B), *H. alpine* (C), and *S. atratum* (D). Schematic illustration of sample types, analysis methods, and specific factors included in this study (E). Regions where the glacier receded ∼10, 70, and 150 years ago (F). Glacier extent adapted from Bruhm et al. (2010).

Rhizosphere samples of three alpine plant species, *P. alpinum, H. alpina*, and *S. atratum*, were collected in the forefield of the Hallstätter glacier (see Fig. 1A–D). We have chosen to sample *H. alpina, P. alpinum*, and *S. atratum*, as they were present in all sampling areas. The plant samples were obtained in regions where the glacier receded ∼10, 70, and 150 years ago; these sampling sites were designated as glacier_10_, glacier_70_, and glacier_150_, respectively (Fig. 1E and F). The sampling followed a long-term permanent plot design initiated by Kühn (2019). Rhizosphere samples were taken by lightly shaking the roots to remove loosely attached soil before they were further treated in the laboratory as described below. The time of deglaciation at the locations was adapted from Bruhm et al. (2010). The mean annual temperature and number of frost-free days at the three sites were obtained using the Climate Downscaling Tool (ClimateDT; https://www.ibbr.cnr.it/climate-dt/, Supplementary Fig. S1A and S1B). At the glacier_10_ sampling site, three independent biological replicates, each consisting of roots with adhering rhizosphere soil from three plants, were obtained from multiple plots. We used homogenized pooled samples from a separately obtained initial sample (*n* = 3 plants per replicate) to acquire a more comprehensive subsample of the microbial community present within the plants. Additionally, bulk soil samples were collected from the area where the glacier receded 10 years ago. However, due to a lack of plants grown in multiple plots at the glacier_70_ and glacier_150_ sampling sites, three biological replicates, with each replicate composed of samples from at least three adjacent plants, were taken from a single plot at the glacier_70_ and glacier_150_ sampling sites. During the sampling event, no bare soil without vegetation could be obtained from the glacier_70_ and glacier_150_ sampling sites. This was also likely attributed to the presence of gravel and small stones as the main soil constituents at the glacier_70_ and glacier_150_ plots. Consequently, it was not possible to compare the microbial data from bulk soil and rhizosphere samples at glacier_70_ and glacier_150_ sampling sites.

To extract DNA from soil and rhizosphere samples, 5 g of plant roots with adhering rhizosphere soil was added to 20 ml of sterile 0.85% NaCl, agitated by hand, and vortexed for 3 min. Samples from aliquots of 2 ml of the obtained suspensions were centrifuged for 20 min at 16 000 × *g* and 4°C in a DuPont Instruments Sorvall RC-5B Refrigerated Superspeed Centrifuge (USA). The resulting pellets were weighed (∼0.1 g) and stored at −20°C until DNA extraction. Total DNA was extracted using the FastDNA Spin Kit for Soil (MP Biomedicals, USA) following the manufacturer’s protocol. Briefly, the pellets were placed in a Lysing Matrix E tube (supplied with the FastDNA™ Spin Kit for Soil) and further processed to lyse microbial cells. The extracted DNA was then purified by a silica-based spin filter method.

### Amplicon sequencing of 16S rRNA genes and shotgun metagenomic sequencing of total community DNA

To investigate potential bacterial functions that may play a role during early succession, we performed shotgun metagenomic sequencing with samples from the sampling sites where the glacier receded 10 years ago (glacier_10_ site, Fig. 1E). The extracted DNA was sent to the sequencing provider Genewiz (Leipzig, Germany). The DNA library preparations and sequencing reactions were performed by the sequencing provider. The DNA sequencing library was prepared using the NEB NextUltra DNA Library Preparation Kit (NEB, UK) according to the guidelines provided by the manufacturer. In brief, the genomic DNA was fragmented using the Covaris S220 instrument and was subjected to end repair and adenylation. Adapters were then ligated following adenylation of the 3′ ends. The adapter-ligated DNA was indexed and enriched by performing limited-cycle polymerase chain reaction (PCR). The DNA sequencing library was then sequenced using an Illumina HiSeq 2500 system and 2 × 150 bp paired-end sequencing.

For all sampling sites, total DNA was subjected to amplicon PCRs to target the whole prokaryotic community (archaea and bacteria, Fig. 1E). We used the 515f/806r primer set to amplify the V4 region of prokaryotic 16S rRNA genes (Caporaso et al. 2012). For demultiplexing, we added sample-specific barcodes to each primer. The barcodes utilized in this study were recommended by the Earth Microbiome Project (http://www.earthmicrobiome.org/). The PCR reaction (25 µl) contained 1 × Taq&Go (MP Biomedicals, Illkirch, France), 0.25 mM of each primer, and 1 µl template DNA. In order to verify the success of the amplification, the PCR products were loaded onto a 1% agarose gel and subjected to gel electrophoresis at 140 V for 60 min. The products were then purified using the Wizard^®^ SV Gel and PCR Clean-Up Kit (Promega, Madison, USA). Subsequently, the DNA concentration of the purified barcoded samples was measured using the Qubit dsDNA BR Assay (Thermo Fischer Scientific) and combined in equal amounts (∼500 ng per sample). The pooled library was sent to the sequencing provider Genewiz (Leipzig, Germany), and the sequencing libraries were prepared using the Nextera XT Index Kit from Illumina. The sequencing libraries were then sequenced using an Illumina MiSeq (v2 reaction kit) instrument with 2 × 300 bp paired-end sequencing.

### Assembly-based metagenomic analyses

Unless otherwise specified, all software were run with the default settings. We used Trimmomatic and VSEARCH to remove Illumina sequencing adaptors and perform preliminary quality filtering on metagenomic reads (removal of low-quality reads; Phred 20). The metagenomic reads were assembled using the Megahit assembler (Li et al. 2015). Only contigs with a length >1 kb were kept for further analysis. The annotation of assembled contigs was conducted using the metagenome classifier Kraken2 (Wood et al. 2019). Open reading frames were predicted using Prodigal v2.6.3 (Hyatt et al. 2010). To remove redundant sequences, we used CD-HIT-EST v4.8.1 to cluster protein-coding gene sequences into a nonredundant gene catalogue using a nucleotide identity of 95% similarity (Li and Godzik 2006). The nonredundant genes were annotated using the blast algorithm in DIAMOND combined with eggNOG-mapper (Buchfink et al. 2015, Huerta-Cepas et al. 2017) and the eggNOG database v5.0 (Huerta-Cepas et al. 2019). We also used eggNOG-mapper to obtain taxonomical assignment for each protein-coding gene. All protein-coding gene sequences that were assigned to *Bacteria* based on the eggNOG-mapper taxonomic classification and with retrievable KEGG Orthology (KO) annotations were kept for further analyses. To generate gene profiles from the samples, we back-mapped quality-filtered reads to the generated nonredundant gene catalogue using BWA and SamTools (Li et al. 2009, Li and Durbin 2010). This step yielded >700 M reads that were classified as bacterial proteins according to the eggNOG mapper.

### Reconstruction of bacterial metagenome-assembled genomes

We used multiple binning methods, i.e. Maxbin2 v2.2.7, MetaBAT2 v2.12.1, and CONCOCT v1.1.0 (Alneberg et al. 2014, Wu et al. 2016, Kang et al. 2019), to construct metagenome-assembled genomes (MAGs). The MAGs with the highest quality among all genome binners were selected using DASTool v1.1.1 (Sieber et al. 2018). Additional binning using Vamb (Nissen et al. 2021) and SemiBin (Pan et al. 2022) was performed using multisample binning approaches by concatenating individual assembled contigs from all samples. The quality of MAGs (completeness and the percentage of contamination) were calculated using CheckM v1.0.13 (Parks et al. 2015). Because we want to compare the metabolic capabilities of different MAGs, only medium-quality MAGs with a completeness >50% and contamination levels <10% according to the current definition of the minimum information MAG standards (Bowers et al. 2017) were kept for further analyses. MAGs were dereplicated using dRep v2.2.3 (Olm et al. 2017) to obtain a nonredundant metagenome-assembled bacterial genome set. We used the Genome Taxonomy Database Toolkit to obtain taxonomical information for each MAG and phylogenetic trees were constructed using PhyloPhlAn (Asnicar et al. 2020) by including closely related taxa from the PhyloPhlAn database. Abundance profiles of each MAG were calculated by using CoverM v0.4.0 (https://github.com/wwood/CoverM) with the option -m rpkm. MAG abundance was calculated as mapped reads per kilobase per million reads divided by the MAG length and total number of reads in each metagenomic dataset (in millions of reads). Gene annotations of constructed MAGs were performed using DRAM v.1.4.6 (Distilled and Refined Annotation of Metabolism) (Shaffer et al. 2020).

### Bacterial community structure and diversity analysis

To analyse the amplicon sequencing dataset, QIIME2 version 2019.10 was used (https://qiime2.org) (Bolyen et al. 2019). Raw reads were demultiplexed and primer sequences were removed using the cutadapt tool (Martin 2011) before importing the data into QIIME2 with the script ‘qiime tools import’. The demultiplexed reads were subjected to quality filtering, denoising, and chimeric sequence removal using the DADA2 algorithm (Callahan et al. 2016). The latter step generated the amplicon sequence variants (ASVs) table, which records the number of times each exact ASV was observed per sample. The output sequences were subsequently aligned against the reference database Silva v132 (Pruesse et al. 2007) using the VSEARCH classifier (Rognes et al. 2016) to obtain taxonomical information of each ASV. In the Silva database, the bacterial class *Betaproteobacteria* was reclassified to the order-level *Betaproteobacteriales* within the bacterial class *Gammaproteobacteria*. Prior to further analyses, only reads assigned to *Bacteria* were retained. Reads assigned to plastids and mitochondria were removed. Negative control used for PCRs produced a minimal number of reads (10 reads—3 ASVs). We eliminated any overlapping ASVs derived from negative controls and excluded the negative control from the datasets. The amplicon sequencing approach resulted in a total of 1 259 583 bacterial reads (min = 4046 and max = 174 837, Supplementary Table S1), which were assigned to a total of 8310 bacterial ASVs.

### Statistical analysis

Bacterial community diversity and composition were analysed in R v4.1.2 using the R packages Phyloseq v1.38.0 and vegan v2.6–4 (Oksanen et al. 2007, R Core Team 2013, McMurdie and Holmes 2013). For alpha diversity analysis, the bacterial abundance table was normalized by subsampling to the lowest number of reads among the samples (4046 reads). The majority of the rarefaction curves obtained for each sample approached the saturation plateau, indicating that the sampling size was sufficient to capture overall bacterial diversity (Supplementary Fig. S1). We estimated alpha diversity using the Shannon index and determined the significance of observed differences using the nonparametric (rank-based) Kruskal–Wallis test, which was followed by a pairwise Wilcox test corrected for multiple comparisons.

MetagenomeSeq’s cumulative sum scaling (CSS) (Paulson et al. 2013) was used for subsequent beta diversity analyses. Beta diversity analysis was performed using a CSS-normalized Bray–Curtis dissimilarity matrix. The dissimilarity matrix was subjected to Adonis analysis to test for significant effects between the different plant species and different regions where the glacier receded. Pairwise Adonis test for multiple comparisons was performed using the pairwiseAdonis v0.4 custom script (https://github.com/pmartinezarbizu/pairwiseAdonis). To investigate the plant specificity of microbial communities, we calculated Spearman correlation coefficients by plotting microbial community dissimilarity between all plant species and different successional ages (10, 70, and 150 years). The relative contribution of deterministic and stochastic processes on bacterial assembly was estimated using the normalized stochasticity ratio (NST) (Ning et al. 2019). Following the randomization of the metacommunity, the NST index was generated using the observed dissimilarity between communities and the randomly expected dissimilarity between communities. The NST index distinguishes between stochastic (>50%) and deterministic (<50%) assemblies. Lastly, linear discriminant analysis and effect size estimation were implemented using LefSe (Segata et al. 2011) to identify bacterial taxa that were enriched in glacier_10_, glacier_70_, and glacier_150_ samples, respectively.

### Identification of enriched ASVs in a global catalogue of microorganisms from various cryospheric ecosytems

We aimed to understand the origins of ASVs that were enriched in glacier_10_, glacier_70_, and glacier_150_ samples. We used a large-scale dataset of the cryosphere (Bourquin et al. 2022) for a deeper analysis to explore whether cryophilic glacier microbes contribute to the soil and plant microbiome of the glacier forefield in our study. Bourquin et al. (2022) generated a global inventory of the microbiome from snow, ice, permafrost soils, and coastal as well as freshwater ecosystems under glacier influence by analysing amplicon sequencing data generated with the same primers as used in our study, 515f-806r targeting prokaryotic 16S rRNA genes. Therefore, using our data, we aligned all the ASVs that were enriched in glacier_10_, glacier_70_, and glacier_150_ samples based on the LefSe analysis with representative sequences from the global catalogue of microorganisms from various cryospheric ecosystems (Bourquin et al. 2022). We assigned ASV matches whether they mapped successfully with 100% coverage and 100% identity against the referred catalogue of 16S rRNA gene–ASVs PP2 (https://doi.org/10.5281/zenodo.6541278).

## Results

### Genome-centric analysis revealed the presence of bacterial key genes for nutrient uptake that can support host plants as well as stress response during early succession

Shotgun metagenome analysis from bulk soil and rhizosphere samples that were collected from area plots the glacier receded 10 years ago allowed us to identify taxa and functions that were enriched in the plant rhizosphere. A gene-centric approach identified a total of 6321 KOs with a maximum relative abundance of 0.46% and a median relative abundance of 0.003% of total mapped reads. We identified genes that might be important for bacteria to survive during early succession. For instance, genes related to manganese and iron transport systems were consistently detected in the metagenome samples (average relative abundance 0.09% of total mapped reads). Cluster genes encode the branched-chain amino acid transporters, *livFGHKM*, which are responsible for the transport of extracellular branched-chain amino acids were detected in high abundance (relative abundance 1.15%). Genes that are associated with chemolithotrophic pathways, i.e. sulfite dehydrogenase and Ni/Fe-hydrogenase were detected (relative abundance 0.04%). A gene encoding for nitrogen fixation (*nifU*) was also recovered from all samples (relative abundance 0.02%). Microbial potential for solubilization and utilization of inorganic phosphate was detected due to the occurrence of genes encoding alkaline phosphatase (*phoA, phoB*, and *phoD*) and inorganic pyrophosphatase (*ppa*). Moreover, we detected genes involved in the production of cold shock proteins (relative abundance 0.89%) and chitinase (relative abundance 0.03%).

**Figure 2.**
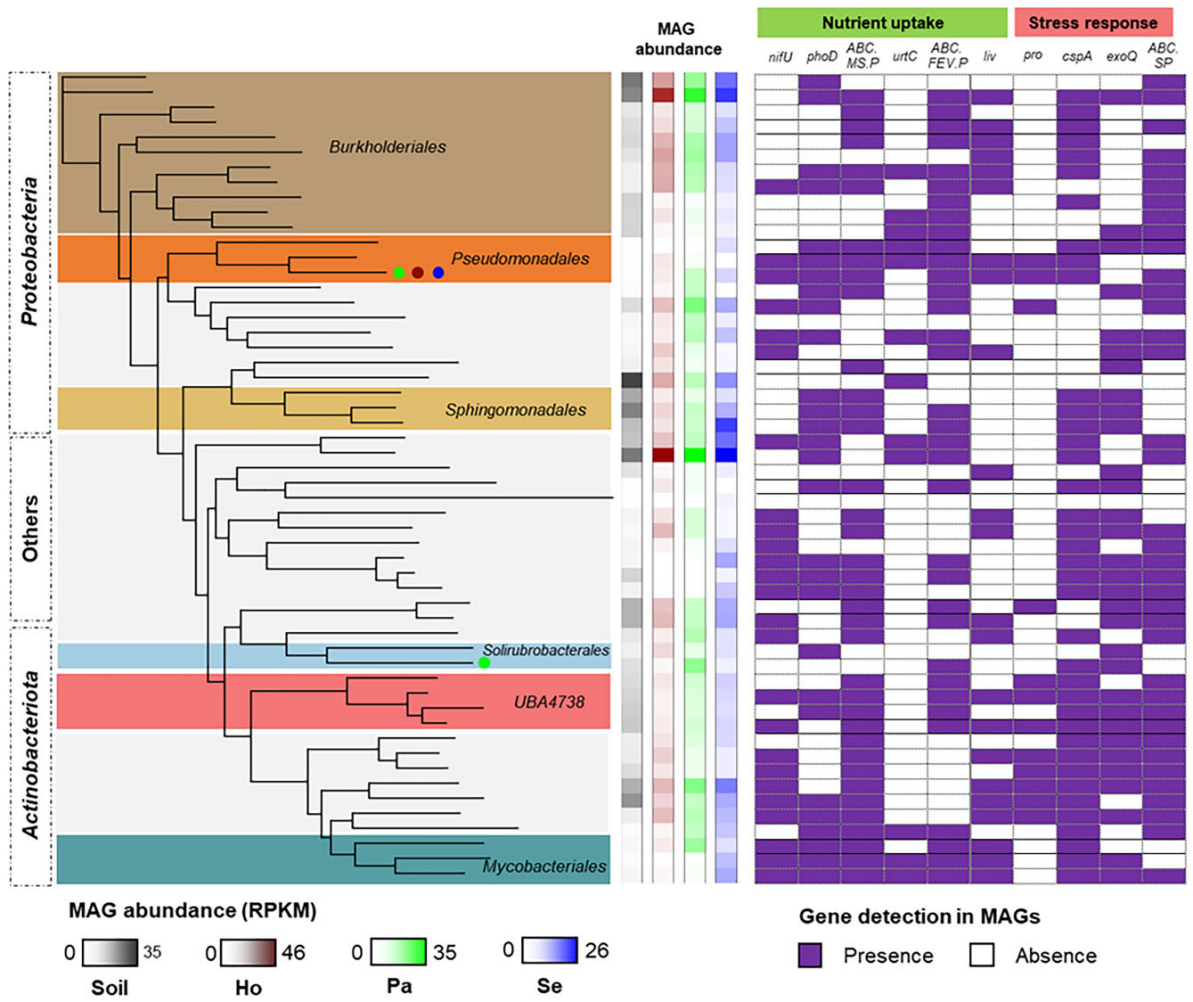
Taxonomic information, abundance, and gene profiles for selected functions in MAGs recovered from the Hallstätter glacier. Analyses were conducted with samples where the glacier receded 10 years ago. Presence (filled)/absence (blank) plots show specific profiles of selected genes in the MAGs. Abundances of MAGs in bulk soil as well as the rhizospheres of *P. alpinum, H. alpine*, and *S. atratum* are based on the number of mapped reads per kilobase per million reads (RPKM) (*n* = 3 replicates).

We further constructed MAGs to compare functional potentials across phylogenetic lineages (Fig. 2). The shotgun metagenomic data yielded a total of 54 bacterial MAGs with a completeness above 50% and contamination levels below 10% (Supplementary Table S2). Among them, six MAGs were considered to represent high-quality genomes (completeness >90% and contamination levels <5%). Most of the MAGs were assigned to *Burkholderiales, Pseudomonadales, Sphingomonadales* (*Proteobacteria*), *Solirubrobacterales, Actinomycetales*, and *Mycobacteriales* (*Actinobacteriota*). MAGs that were assigned to the bacterial orders *Pseudomonadales, Steroidobacterales, Actinomycetales, Mycobacteriales*, and *SG8-23* carried the *nifU* gene that encodes a nitrogen fixation protein. More than half of MAGs carried *phoD*, encoding an alkaline phosphatase. This gene could also be detected within different bacterial orders such as *Burkholderiales, Pseudomonadales, Sphingomonadales, Actinomycetales*, and *Mycobacteriales*. Several genes that encode proteins related to nutrient uptake, i.e. multiple sugar transport system (*ABC.MS.P*), urea transport system (*urtC*), and iron complex transport system (*ABC.FEV.P*) were found. Among the MAGs, we detected high occurrences of genes encoding a cold shock protein (*cspA*), an exopolysaccharide production protein (*exoQ*), and a spermidine/putrescine transport system (*ABC.SP.P*) that are likely related to the adaptability and stress response of bacteria in the Hallstätter glacier.

### Similar bacterial functions were detected in the rhizosphere of different plants and soil samples during early succession

We detected a highly similar gene profile between rhizosphere samples of different plant species. Adonis analysis indicated no difference in bacterial gene functional profiles between different plant species that grew where the glacier receded 10 years ago (Adonis—*P* = 0.298). When the bulk soil samples were included, the gene profiles of the rhizosphere samples were significantly different in comparison to them (Adonis—*P* = 0.028, R^2^ = 43%). However, pairwise analysis only indicated a certain tendency for the presence of different gene profiles between bulk soil and rhizosphere of *P. alpinum* and *H. alpina* (*P* = 0.100), while no difference was observed when compared to *S. atratum* samples (*P* = 0.200). Overall, only minor differences in gene profiles between the rhizospheres of different plants and the bulk soil samples were observed during the early succession represented by glacier_10_ samples.

Pairwise comparisons between rhizosphere samples from different plants and bulk soil samples suggested only minor differences in microbial functioning during the early succession event. Of the 6321 detected KO, a closer look at the differentially abundant functions identified only a small number of KOs that were enriched (n_KO_) in the rhizosphere samples of *H. alpine* (*n*_KO_ = 23), *S. atratum* (*n*_KO_ = 26), and *P. alpinum* (*n*_KO_ = 1) when compared to bulk soil samples (LefSe—*P* < 0.05, LDA score > 2). Therein, we detected genes that encode glutamate synthase (*GLU*), branched-chain amino acid transport systems (*livK* and *livM*), serine/threonine-protein kinase (*prkC*), malate dehydrogenase (*maeB*), and heavy metal efflux transporter (*czcA*).

### Shifts in rhizosphere bacterial richness and community structure during primary colonization of the Hallstätter glacier forefield

**Figure 3.**
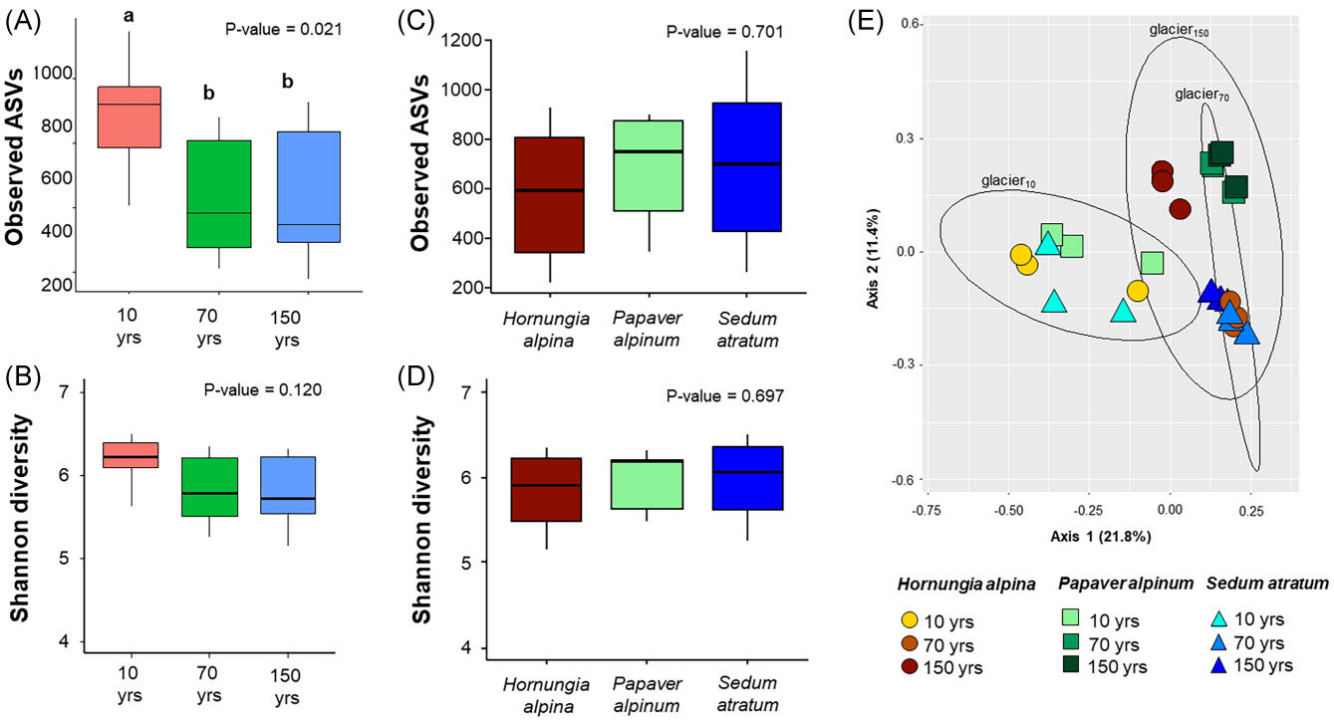
Bacterial richness and community structure in the plant rhizosphere along the deglaciation chronosequence. Bacterial richness (A and B) and diversity (C and D) in the rhizosphere were determined for different sampling areas (glacier_10_, glacier_70_, and glacier_150_) and for different plant species. Data are presented as the mean for each group (*n* = 3 replicates). A principal coordinate analysis (PCoA) plot was used to visualize the clustering of bacterial community structures (E). Standard error ellipses indicate 95% confidence areas.

Using the amplicon sequencing dataset, we explored microbial succession in the rhizosphere of the three plant species, *H. alpina, P. alpinum*, and *S. atratum* along the deglaciation chronosequence. Amplicon sequencing resulted in a congruent result, as observed from shotgun metagenome data. Bulk soil samples clustered together with rhizosphere samples from the glacier front (glacier_10_, Supplementary Fig. S2), indicating high similarity between these samples. To investigate the impact of deglaciation and host plants on the bacterial richness and community structures, soil samples were excluded from the analysis. Deglaciation affected bacterial richness (Kruskal–Wallis test—*P* = 0.021, Fig. 3A) but not bacterial diversity in the rhizosphere (*P* = 0.120, Fig. 3B). A higher bacterial richness (*n*_ASV_ = 875) was found for glacier_10_ in comparison to other regions (glacier_70–_*n*_ASV_ = 556; glacier_150–_*n*_ASV_ = 544). In contrast, a significant difference in bacterial richness and diversity was not observed when plant species were used as factors (Kruskal–Wallis test—*P* = 0.701 and *P* = 0.697, Fig. 3C and D).

Shifts in the bacterial community composition in the rhizosphere as a response to deglaciation were observed. The constructed PcoA plot indicated that samples were clustered according to the deglaciation period. All samples that were obtained from bulk soil near the glacier front (glacier_10_) showed a tendency to cluster together (Fig. 3E). Adonis analysis indicated that deglaciation contributed significantly to bacterial community variations (*P* = 0.001, R^2^ = 28%). Furthermore, according to the PcoA plot, the rhizosphere microbiome of *P. alpinum* from glacier_70_ and glacier_150_ samples clustered together. A similar pattern was observed for the rhizosphere microbiome of *S. atratum*, whereas the rhizosphere microbiomes of *H. alpina* obtained from the glacier_70_ and glacier_150_ locations ordinated away from each other. Plant species also contributed significantly to the bacterial community variations but to a lesser degree (Adonis—*P* = 0.001, R^2^ = 14%). When analysed separately for each deglaciation region, we did not observe a significant difference in rhizosphere bacterial community composition between the different plant species that were obtained from glacier_10_ (Adonis—*P* = 0.366, R^2^ = 26%). This result reflects the nonsignificant differences in bacterial functional profiles in the rhizosphere of different plant species that grew within the glacier_10_ site, as described previously. However, significant differences in bacterial community composition between plant species were observed in the samples obtained from glacier_70_ (Adonis—*P* = 0.007, R^2^ = 60%) and glacier_150_ (Adonis—*P* = 0.005, R^2^ = 61%).

When calculating bacterial community dissimilarity between different plant species, we observed a higher bacterial community dissimilarity between different plant species at the later stages of the succession rather than the early stage. Additionally, Spearman’s correlation analysis indicated that bacterial community dissimilarity between different plant species was positively correlated to successional age (*P* = 0.009, r = 0.35; Supplementary Fig. S3A). Moreover, our results showed that bacterial community assembly was more stochastic at glacier_10_ (NST = 64%) compared to glacier_70_ (NST = 51%—Wilcoxon test *P* < 0.001) and glacier_150_ (NST = 47%—Wilcoxon test *P* < 0.001) (Supplementary Fig. S3B). Taken together, these results suggest that bacterial communities were potentially selected by the plant species at later stages of the succession (i.e. glacier_70_ and glacier_150_).

**Figure 4.**
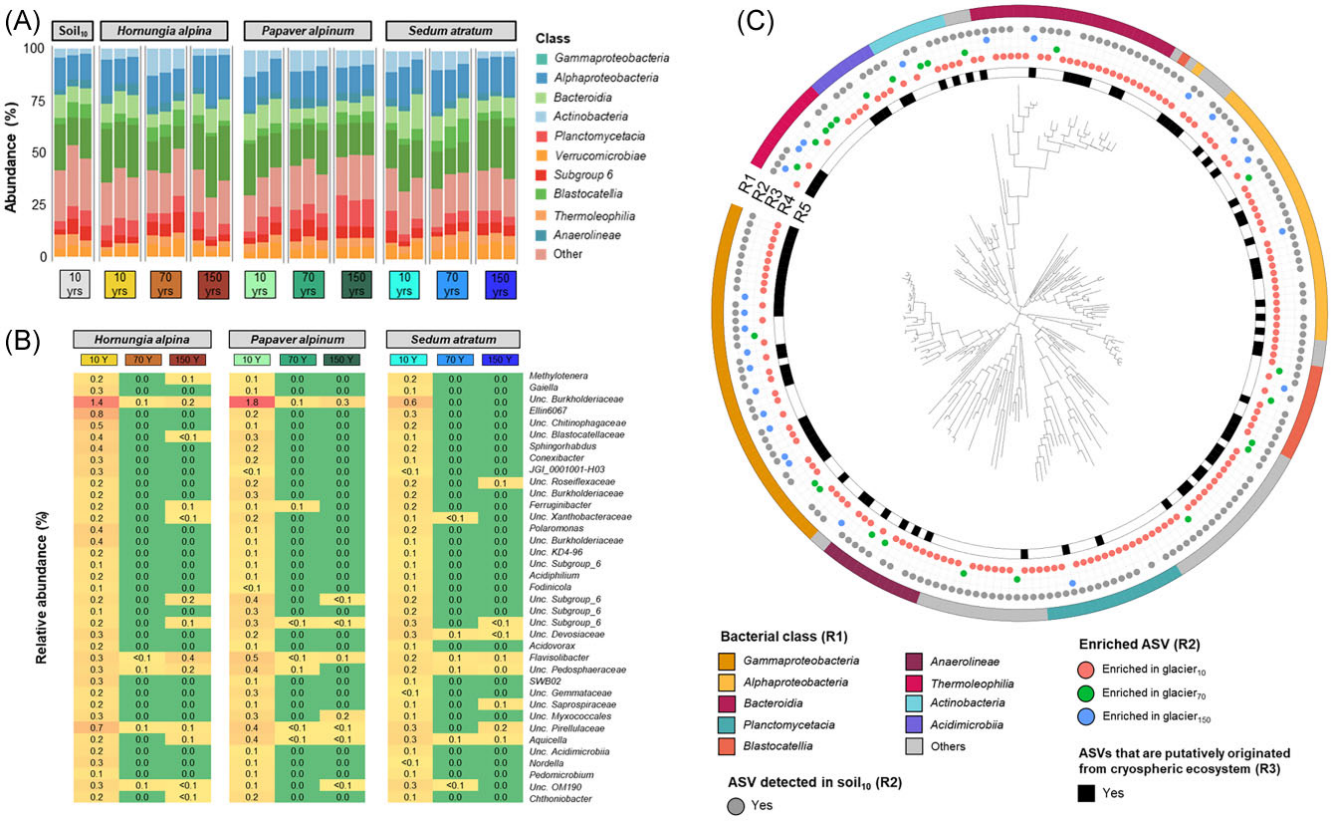
Bacterial community composition and taxa that were enriched in the plant rhizosphere from different sampling areas_._ Bacterial composition is shown at the class level (A). LEfSe analysis revealed specific biomarkers at the bacterial ASV level (B). Only bacterial ASVs that were enriched in the rhizosphere from glacier_10_ samples with LDA scores > 2.8 and *P*_adjusted_ < 0.05 according to LEfSe analysis are shown. Phylogenetic tree based on partial 16S rRNA genes of ASVs enriched between sites where the glacier receded 10, 70, and 150 years ago (C). Ring 1 (R1) indicates bacterial taxonomy. Ring 2 (R2) indicates ASVs that were found in bulk soil from the glacier_10_ area. Ring 3 (R3) indicates the area where the ASVs were enriched. Ring 4 (R4) indicates if the ASVs that were enriched shared a high similarity to the representative sequences from the global catalogue of microorganisms from various cryospheric ecosystems (Bourquin et al. 2022).

*Gammaproteobacteria, Alphaproteobacteria, Bacteroidia*, and *Actinobacteria* were identified as the most abundant bacterial classes, which contributed to 19.6%, 15.0%, 9.0%, and 6.6% of the total relative abundance, respectively (Fig. 4A). We did not observe gradual changes in the relative abundance of the two dominant bacterial classes, i.e. *Gammaproteobacteria* and *Alphaproteobacteria* for different deglaciation periods. *Gammaproteobacteria* were the dominant bacterial class in *H. alpina* (22.7% and 26.5%) and *S. atratum* (20.3% and 23.7%) in glacier_10_ and glacier_150_ samples, respectively (Fig. 4A). Interestingly, the relative abundance of *Actinobacteria* (4.0%—glacier_10_, 11.6%—glacier_70_, 3.2%—glacier_150_) in the rhizosphere of *H. alpina* and the relative abundance of *Blastocatellia* (3.9%—glacier_10_, 9.5%—glacier_70_, 3.9%—glacier_150_) in the rhizosphere of *S. atratum* showed an opposite pattern. The relative abundance of *Actinobacteria* was relatively low in the bulk soil obtained from glacier_10._ Relative abundances of *Alphaproteobacteria* were relatively stable for the different deglaciation periods (*H. alpina–*14.1%–19.7%, *P. alpinum*–12.2%–15.1%, and *S. atratum*–16.3%–17.5%). Relative abundances of *Bacteroidia* were higher in the rhizosphere of all plant species as well as bulk soil samples that were collected in the area of early succession, where the glacier receded 10 years ago, when compared to other areas. For instance, the relative abundance of *Bacteroidia* was lower in the rhizosphere of *P. alpinum* collected in the region where the glacier receded 70 and 150 years ago (6.6% and 6.1%, respectively, Fig. 4A), compared to the region where the glacier receded 10 years ago (11.3%). The same pattern was observed for the rhizosphere bacterial community of *S. atratum* (Fig. 4A).

We performed differential abundance analysis at the ASV level using LefSe and identified 212 bacterial ASVs that were differentially abundant between the sites where the glacier receded 10, 70, and 150 years ago. Of these ASVs, the relative abundance of 164 ASVs decreased in glacier_70_ and glacier_150_ samples in comparison to glacier_10_ samples. The majority of these ASVs (*n* = 112) were undetectable in the rhizosphere of all plant species that grew at the glacier_70_ and glacier_150_ locations (Fig. 4B and C). Most of these ASVs belonged to the bacterial orders *Betaproteobacteriales* (*n* = 22), *Chitinophagales* (*n* = 19), *Rhizobiales* (*n* = 10), *Gemmatales* (*n* = 8), and *Blastocatellales* (*n* = 7) (Fig. 4C). The ASVs enriched in the rhizosphere of all plant species that grew at the glacier_10_ were also found in bulk soil collected from the glacier_10_ location (Fig. 4C), indicating that the surrounding soil was the main reservoir of bacteria that colonized the rhizosphere of all plant species that grew at the glacier_10_. Interestingly, a total of 67 ASVs that were enriched in the sites where the glacier receded 10 years ago had a high similarity (100% identity and 100% sequence coverage) with ASVs found in various cryospheric ecosystems (Fig. 4C). In contrast, only 24 ASVs were enriched in glacier_70_ as well as glacier_150_ samples, respectively. These ASVs belonged to *Betaproteobacteriales* (*n* = 9), *Solirubrobacterales* (*n* = 4), *Pseudonocardiales* (*n* = 2), *Chitinophagales* (*n* = 2), and *Xanthomonadales* (*n* = 2). These results indicate that ASVs that were enriched in the sites where the glacier receded 10 years ago likely originated from the glacier.

## Discussion

Our study on plant-associated bacterial communities during early succession in the forefield of the Hallstätter glacierprovides novel insights into the temporal dimension of the assembly of the plant rhizosphere microbiota. We found that bacterial genes encode potential functional adaptations to the glacier environment. The rhizosphere microbiomes of the alpine plants *H. alpina, P. alpinum*, and *S. atratum* showed clear differences along the chronosequence. These differences were characterized by decreasing microbial richness but increasing specificity of plant-associated bacterial communities in the rhizosphere. Altogether, the findings indicate that time plays a significant role in the assembly of the rhizosphere bacterial communities across the chronosequence. These bacteria potentially support pioneer plants in the process of colonizing new habitats and their long-term establishment at later stages.

When microbial functions were analysed at the glacier_10_ site, our data indicated key features related to bacterial adaptation to the glacier forefield. *Betaproteobacteria, Gammaproteobacteria, Alphaproteobacteria*, and *Actinobacteria* dominated the forefield of the glacier. *Burkholderiales* (*Betaproteobacteria*), *Sphingomonadales* (*Alphaproteobacteria*), *Micrococcales*, and *Mycobacteriales* (*Actinobacteria*) were previously found in other regions with low mean annual temperatures, e.g. the Damma glacier (Lapanje et al. 2012), the glacial region of Sikkim Himalaya (Mukhia et al. 2022), and the Svalbard glacier (Perini et al. 2019, Tian et al. 2022). The low temperatures and frequent temperature fluctuations around the freezing point, which can cause cold shock responses in microbial cells, are common in harsh alpine habitats. By coupling short-read-based and genome-centric analyses, we provided evidence that certain bacterial taxa in the deglaciated area are functionally adapted to cold temperatures and limited nutrients due to the occurrence of genes encoding cold shock proteins and nutrient uptake. The presence of genes encoding a particular cold shock protein, i.e. *cspA*, is crucial to maintain protein homeostasis during cold stress (Xia et al. 2001, Kumar et al. 2020). Moreover, genes that are involved in the production of exopolysaccharides (*exo* genes) and the spermidine/putrescine transport system are important to protect bacteria from abiotic stresses, i.e. drought stress and cold stress (Naseem et al. 2018, Morcillo and Manzanera 2021), and play important roles in root colonization (Liu et al. 2020). Microbial exopolysaccharides also cause soil particle aggregation, which is important for soil structure formation and the accumulation of nutrients (Costa et al. 2018). During early succession and especially in cold regions, soil is dominated by mineral phosphate, which is highly insoluble and not available for plants (Heindel et al. 2017, Ren et al. 2022). Hence, genes encoding alkaline phosphatases (encoded by *phoA, phoB*, and *phoD*) are likely needed for bacteria to increase phosphate availability under phosphorus-limited conditions. Furthermore, the occurrence of genes encoding multiple sugar, iron, and branched-chain amino acid transport systems may provide a benefit to scavenge and access resources from the surrounding environment.

The succession stage in the Hallstätter glacier forefield had a substantial impact on the microbial community in the rhizosphere of pioneer plants. A recent study by Mapelli *et al*. (2018) examined changes in bacterial diversity in the rhizosphere of a pioneer plant along a High Arctic glacier chronosequence. The authors observed that changes of total nitrogen, total organic carbon, and cation exchange capacity during the developmental stage of the soil strongly affect the bacterial community in the rhizosphere throughout the chronosequence. Interestingly, the bacterial community functions and structure in the rhizosphere did not differ significantly between different plants at the glacier_10_ site. During the initial stages of succession, the abiotic factors present in the studied Hallstätter glacier pose challenging conditions for microbial survival. The identified microorganisms exhibit common characteristics that enable them to adapt and endure the adverse environmental conditions. Based on our findings, it can be inferred that during early succession, i.e. the glacier_10_ region, different plant species recruit similar microbes from the surrounding soil, which are ubiquitous and well adapted to this particular environment. Moreover, the specific environment in the glacier_10_ region, especially due to reduced frost-free days, could also limit the ability of the host plant to shape the bacterial community structure and functioning in the rhizosphere.

In this study, from the site closest to the glacier to the older sites, the rhizosphere microbial community showed an increase in host specificity, but a decrease in rhizosphere microbial richness. Interestingly, we observed that several bacterial ASVs present at the glacier_10_ were undetectable at the glacier_70_ and glacier_150_ locations. These taxa may have partially originated from the glacier, as indicated by the high similarity with bacterial sequences from various cryospheric ecosystems, and might therefore be sensitive to changing habitat conditions that occurred at the glacier_70_ and glacier_150_ locations. Moreover, we argue that host plants only maintain certain taxa that provide ecological services and functional traits necessary for promoting fitness and resilience of the host at later stages of succession. It is widely acknowledged that the plant host is one of the main drivers of rhizosphere microbiome assembly (Berg and Smalla 2009, Hassani et al. 2018). The selection of the rhizosphere community by host plants is based on functional features related to plant metabolism (Mendes et al. 2014). After the early succession stage, with nutrients becoming more available and more days without frost, the production of root exudates that select specific rhizosphere bacteria is likely more pronounced_._ Improved specificity during assembly of rhizosphere microbial communities is also indicated by the observed decrease in stochasticity and was previously described for plant communities. Our findings are in line with a recent study by Hanusch et al. (2022) that suggested environmental filtration and biotic interactions replace stochasticity after 60 years of succession in a glacier forefied. Despite conducting a thorough analysis, this study has certain limitations. These include a relatively limited number of biological replicates and a restricted number of sampling plots where the studied plant species were naturally growing. Moreover, soil chemistry data such as soil pH, water content, and organic matter content during soil development, which could potentially impact bacterial community structures, were not considered in this study. These limitations emphasize the importance of conducting future research with a larger sample size and including these relevant factors in order to validate and confirm the impact of deglaciation on bacterial community structures.

In conclusion, we revealed that the Hallstätter glacier is a source of specific, cold-adapted bacterial communities, which are likely diminished during deglaciation. While plant-specific microorganisms facilitate long-term establishment, well-adapted ubiquitous bacteria from surrounding soil may allow pioneer plants to colonize new habitats. This pattern was reflected by a decrease in bacterial richness but an increase in specificity in plant-associated bacterial community in the rhizosphere along the gradient of deglaciation.

## Supplementary Material

fiae005_Supplemental_File

## Data Availability

The sequencing data have been deposited in the European Nucleotide Archive (ENA) database under the study number PRJEB63480 (amplicon sequencing dataset) and PRJEB63481 (shotgun metagenome dataset).
