# Supplementary material for: Rhizosphere assembly alters along a chronosequence in the Hallstätter glacier forefield (Dachstein, Austria)
Source: FEMS Microbiol Ecol. 2024 Jan 25;100(2):fiae005. doi: 10.1093/femsec/fiae005 (PMC10858390; doi:10.1093/femsec/fiae005)
Supplement: fiae005_Supplemental_File [file fiae005_supplemental_file.docx]

**Supplementary Material**

**Rhizosphere assembly alters along a chronosequence in the Hallstätter glacier forefield (Dachstein, Austria)**

**Wisnu Adi Wicaksono^a^, Max Mora^a^, Samuel Bickel^a^, Christian Berg^b^, Ingolf Kühn^cde^**, **Tomislav Cernava^a^, Gabriele Berg^afg^**

*^a^Institute of Environmental Biotechnology, Graz University of Technology, Graz, Austria*

*^b^Institute of Plant Sciences, University of Graz, Graz, Austria*

*^c^Helmholtz Centre for Environmental Research - UFZ, Department of Community Ecology, Halle, Germany*

*^d^Institute of Biology/Geobotany and Botanical Garden, Martin Luther University Halle‐Wittenberg, Halle, Germany*

*^e^German Centre for Integrative Biodiversity Research (iDiv) Halle-Jena-Leipzig, Leipzig, Germany*

*^f^Leibniz-Institute for Agricultural Engineering and Bioeconomy Potsdam (ATB), Potsdam, Germany*

*^g^Institute for Biochemistry and Biology, University of Potsdam, Potsdam, Germany*

^#^Corresponding authors:

Wisnu Adi Wicaksono and Gabriele Berg, Graz University of Technology, Graz

Emails: [wisnu.wicaksono@tugraz.at](mailto:wisnu.wicaksono@tugraz.at), & gabriele.berg@tugraz.at[:](mailto:)

**Authors’ email addresses**

maxmora.go@gmail.com, samuel.bickel@tugraz.at, christian.berg@uni-graz.at, ingolf.kuehn@ufz.de, tomislav.cernava@tugraz.at

Submitted to: FEMS Microbiology Ecology


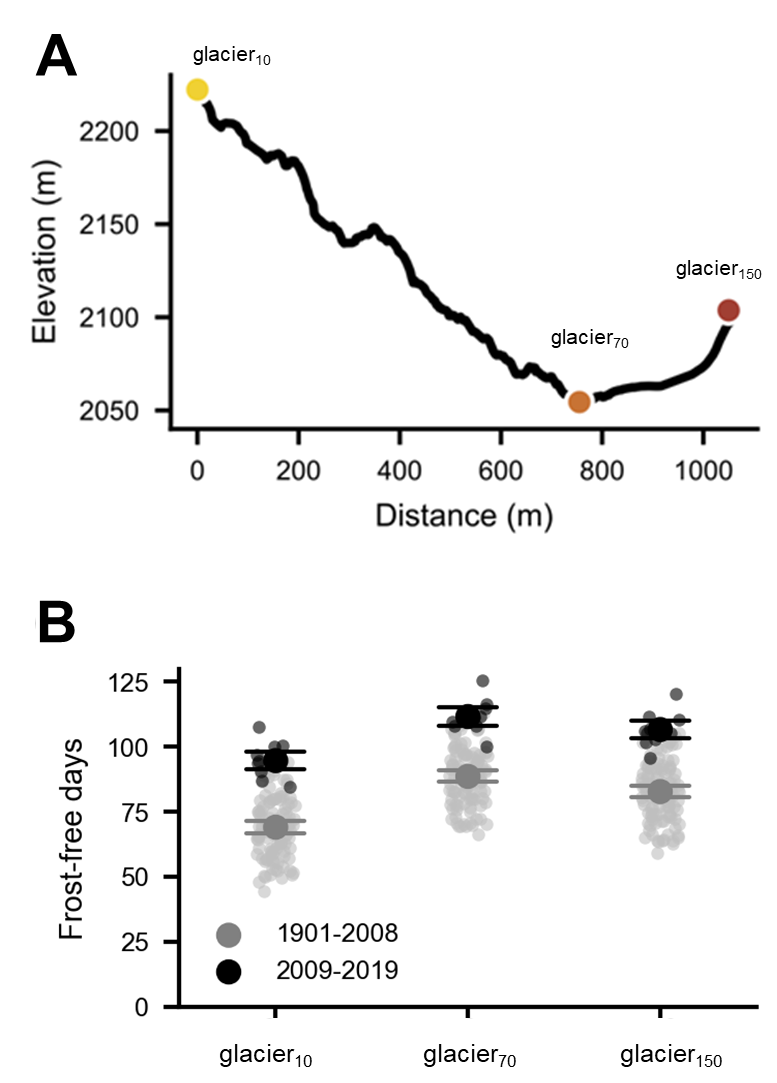


**Supplementary Figure S1.** Distance of regions where the glacier receded approximately 10, 70, and 150 years ago elevation (A), and frost-free days (B) between sampling sites. Glacier extent adapted from Bruhm et al., (2010). Source of the orthophoto and elevation data: Land Oberösterreich - data.ooe.gv.at.

**Supplementary Figure S2. Rarefaction curves showing the number of reads that were classified as bacterial sequences from the rarefied dataset.**

**
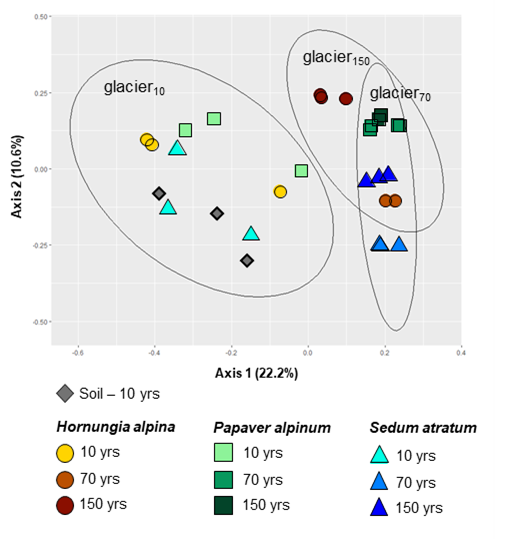
**

**Figure S3. Bacterial community structure in soil and rhizosphere**. A principal coordinate analysis (PCoA) plot was used to visualize the clustering of bacterial community structures. Standard error ellipses indicate 95% confidence areas.


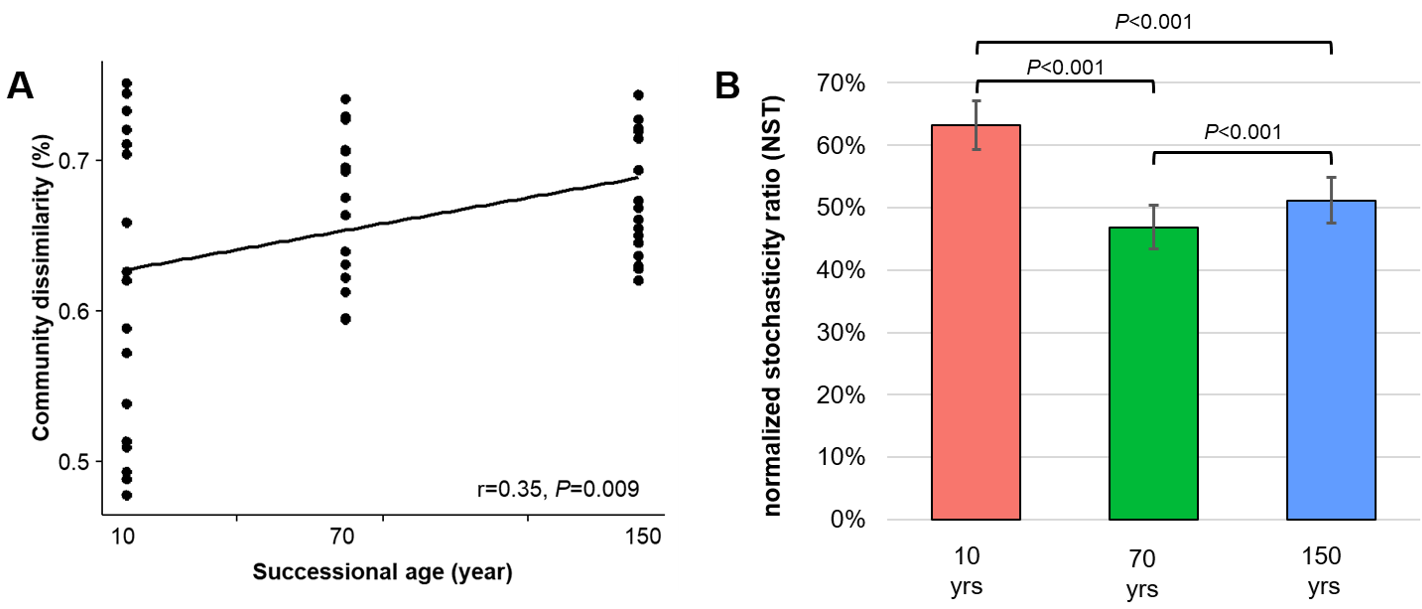


**Supplementary Figure S4. Correlation analysis (A) between bacterial community dissimilarity between different plant species and successional age (glacier_10_, glacier_70_ and glacier_150_) and the barplot of the normalized stochasticity ratio (NST) index based on Bray–Curtis distance (B).**

| Sample ID | High-quality reads | Non-bacterial reads | Bacterial reads |
| --- | --- | --- | --- |
| Soil1 | 131568 | 12291 | 119277 |
| Soil2 | 108153 | 6633 | 101520 |
| Soil3 | 88851 | 657 | 88194 |
| Ho_10_1 | 84377 | 1111 | 83266 |
| Ho_10_2 | 55729 | 3414 | 52315 |
| Ho_10_3 | 93180 | 1474 | 91706 |
| Ho_70_1 | 10799 | 815 | 9984 |
| Ho_70_2 | 4610 | 564 | 4046 |
| Ho_70_3 | 5658 | 453 | 5205 |
| Ho_150_1 | 22580 | 274 | 22306 |
| Ho_150_2 | 8044 | 44 | 8000 |
| Ho_150_3 | 25811 | 383 | 25428 |
| Pa_10_1 | 26042 | 1034 | 25008 |
| Pa_10_2 | 65652 | 1027 | 64625 |
| Pa_10_3 | 18720 | 62 | 18658 |
| Pa_70_1 | 8566 | 1323 | 7243 |
| Pa_70_2 | 37218 | 3080 | 34138 |
| Pa_70_3 | 33114 | 2257 | 30857 |
| Pa_150_1 | 23363 | 1619 | 21744 |
| Pa_150_2 | 23742 | 1539 | 22203 |
| Pa_150_3 | 7323 | 447 | 6876 |
| Se_10_1 | 72309 | 3951 | 68358 |
| Se_10_2 | 176656 | 1819 | 174837 |
| Se_10_3 | 96820 | 1398 | 95422 |
| Se_70_1 | 29315 | 4028 | 25287 |
| Se_70_2 | 24180 | 2327 | 21853 |
| Se_70_3 | 11260 | 1610 | 9650 |
| Se_150_1 | 10843 | 175 | 10668 |
| Se_150_2 | 6704 | 196 | 6508 |
| Se_150_3 | 4494 | 93 | 4401 |

**Supplementary** Table S1 **Number of amplicon sequencing reads**

**Supplementary** Table S2 **Detailed taxonomic classification, completeness and contamination values of bacterial metagenome assembled genomes**

| GenomeID | Completeness / Contamination (%) | Taxonomical information |
| --- | --- | --- |
| DS1 | 54.62 / 4.61 | Proteobacteria;Gammaproteobacteria;GCA-2729495;GCA-2729495;-; |
| DS2 | 67.99 / 0.06 | Eisenbacteria;RBG-16-71-46;RBG-16-71-46;RBG-16-71-46;WS-11; |
| DS3 | 63.56 / 1.96 | Proteobacteria;Gammaproteobacteria;Burkholderiales;SG8-40;-; |
| DS4 | 64.81 / 1.06 | Proteobacteria;Gammaproteobacteria;Burkholderiales;Nitrosomonadaceae;Nitrosospira; |
| DS5 | 98.9 / 1.1 | Eisenbacteria;RBG-16-71-46;SZUA-252;SZUA-252;SZUA-252; |
| DS6 | 53.69 / 8.37 | Proteobacteria;Gammaproteobacteria;Burkholderiales;SG8-39;RBG-16-66-20; |
| DS7 | 56.96 / 4.39 | Proteobacteria;Gammaproteobacteria;Burkholderiales;UKL13-2;GR16-43; |
| DS8 | 79.67 / 9.83 | Actinobacteriota;Thermoleophilia;Solirubrobacterales;-;-; |
| DS9 | 94.83 / 6.27 | Actinobacteriota;Actinomycetia;Actinomycetales;-;-; |
| DS10 | 54.68 / 7.26 | Proteobacteria;Alphaproteobacteria;Acetobacterales;Acetobacteraceae;-; |
| DS11 | 50.9 / 7.97 | Proteobacteria;Alphaproteobacteria;Rhodobacterales;Rhodobacteraceae;-; |
| DS12 | 63.95 / 6.48 | Chloroflexota;Ellin6529;CSP1-4;CSP1-4;SPCO01; |
| DS13 | 66.19 / 5.43 | Proteobacteria;Gammaproteobacteria;Steroidobacterales;Steroidobacteraceae;SCUD01; |
| DS14 | 77.33 / 7.31 | Actinobacteriota;Actinomycetia;Actinomycetales;Micrococcaceae;Pseudarthrobacter;Pseudarthrobacter sp002929755 |
| DS15 | 56.58 / 1.54 | Proteobacteria;Gammaproteobacteria;Burkholderiales;Burkholderiaceae;-; |
| DS16 | 88.48 / 3.27 | Actinobacteriota;Actinomycetia;Mycobacteriales;Nakamurellaceae;Nakamurella; |
| DS17 | 61.54 / 6.66 | Proteobacteria;Gammaproteobacteria;Burkholderiales;SG8-40;-; |
| DS18 | 87.02 / 1.23 | Actinobacteriota;Actinomycetia;Mycobacteriales;Micromonosporaceae;Dactylosporangium; |
| DS19 | 58.49 / 3.98 | Proteobacteria;Gammaproteobacteria;Steroidobacterales;Steroidobacteraceae;UBA964; |
| DS20 | 78.62 / 6.9 | Proteobacteria;Gammaproteobacteria;Burkholderiales;Burkholderiaceae;JOSHI-001; |
| DS21 | 69.55 / 2.65 | Planctomycetota;Phycisphaerae;Phycisphaerales;SM1A02;-; |
| DS22 | 96.04 / 2.05 | Actinobacteriota;Actinomycetia;Actinomycetales;Dermatophilaceae;UBA4719; |
| DS23 | 61.21 / 0 | Proteobacteria;Gammaproteobacteria;Pseudomonadales;Pseudomonadaceae;Pseudomonas_E;Pseudomonas_E brenneri_B |
| DS24 | 68.26 / 4.47 | Proteobacteria;Gammaproteobacteria;Pseudomonadales;Pseudomonadaceae;Pseudomonas_E;Pseudomonas_E sp900187425 |
| DS25 | 62.77 / 4 | Actinobacteriota;Thermoleophilia;Gaiellales;Gaiellaceae;GMQP-bins7; |
| DS26 | 89.13 / 3.25 | Proteobacteria;Gammaproteobacteria;Steroidobacterales;Steroidobacteraceae;-; |
| DS27 | 50.86 / 0 | Actinobacteriota;Thermoleophilia;Solirubrobacterales;Solirubrobacteraceae;-; |
| DS28 | 56.56 / 5.56 | Gemmatimonadota;Gemmatimonadetes;Gemmatimonadales;GWC2-71-9;SPDF01; |
| DS29 | 84.57 / 3.85 | Gemmatimonadota;Gemmatimonadetes;SG8-23;SKRI01;-; |
| DS30 | 73.4 / 2.66 | Actinobacteriota;UBA4738;UBA4738;HRBIN12;-; |
| DS31 | 82.7 / 6.95 | Actinobacteriota;Actinomycetia;Propionibacteriales;Nocardioidaceae;Marmoricola; |
| DS32 | 85 / 2.43 | Proteobacteria;Gammaproteobacteria;Burkholderiales;Burkholderiaceae;-; |
| DS33 | 93.41 / 4.95 | Gemmatimonadota;Gemmatimonadetes;SG8-23;SKRI01;-; |
| DS34 | 58.61 / 0.52 | Patescibacteria;Saccharimonadia;Saccharimonadales;UBA4665;PMNU01; |
| DS35 | 93.68 / 1.16 | Actinobacteriota;Actinomycetia;Mycobacteriales;Micromonosporaceae;-; |
| DS36 | 57.66 / 0.24 | Proteobacteria;Gammaproteobacteria;Pseudomonadales;UBA7239;-; |
| DS37 | 90.05 / 5.04 | Proteobacteria;Alphaproteobacteria;Sphingomonadales;Sphingomonadaceae;Allosphingosinicella; |
| DS38 | 78.12 / 9.75 | Nitrospirota;Nitrospiria;Nitrospirales;Nitrospiraceae;Nitrospira; |
| DS39 | 50.78 / 9.93 | Actinobacteriota;UBA4738;UBA4738;HRBIN12;-; |
| DS40 | 57.75 / 8.71 | Actinobacteriota;Actinomycetia;Actinomycetales;-;-; |
| DS41 | 57 / 7.68 | Nitrospirota;Nitrospiria;Nitrospirales;Nitrospiraceae;Palsa-1315; |
| DS42 | 87.44 / 1.03 | Actinobacteriota;UBA4738;UBA4738;HRBIN12;-; |
| DS43 | 59.84 / 1.38 | Actinobacteriota;Actinomycetia;Propionibacteriales;Nocardioidaceae;Nocardioides; |
| DS44 | 67.01 / 6.65 | Actinobacteriota;Actinomycetia;Sporichthyales;Sporichthyaceae;-; |
| DS45 | 51.41 / 0.46 | Chloroflexota;Ellin6529;CSP1-4;CSP1-4;SPCO01; |
| DS46 | 62.3 / 5.56 | Proteobacteria;Alphaproteobacteria;Sphingomonadales;Sphingomonadaceae;LB1R16; |
| DS47 | 65.63 / 8.65 | Proteobacteria;Gammaproteobacteria;Burkholderiales;Burkholderiaceae;Methylibium; |
| DS48 | 99.5 / 2.75 | Proteobacteria;Alphaproteobacteria;Sphingomonadales;Sphingomonadaceae;Allosphingosinicella; |
| DS49 | 55.93 / 1.98 | Proteobacteria;Gammaproteobacteria;Burkholderiales;Nitrosomonadaceae;Nitrosospira; |
| DS50 | 51.67 / 0.03 | Proteobacteria;Gammaproteobacteria;Burkholderiales;Gallionellaceae;Nitrotoga; |
| DS51 | 58.68 / 5.15 | Actinobacteriota;UBA4738;UBA4738;HRBIN12;-; |
| DS52 | 90.11 / 3.85 | Gemmatimonadota;Gemmatimonadetes;SG8-23;SKRI01;-; |
| DS53 | 57.03 / 8.47 | Proteobacteria;Gammaproteobacteria;UBA6522;UBA6522;FEN-1219; |
| DS54 | 53.49 / 4.27 | Acidobacteriota;Thermoanaerobaculia;UBA5066;Gp7-AA6;Gp7-AA6; |
